# Endolysosomal transient receptor potential mucolipins and two-pore channels: implications for cancer immunity

**DOI:** 10.3389/fimmu.2024.1389194

**Published:** 2024-05-22

**Authors:** Lina Ouologuem, Karin Bartel

**Affiliations:** Department of Pharmacy, Drug Delivery, Ludwig-Maximilians-University Munich, Munich, Germany

**Keywords:** lysosome, ion channels, TRPML, TPC, cancer, cancer immunity, two-pore channels, mucolipins

## Abstract

Past research has identified that cancer cells sustain several cancer hallmarks by impairing function of the endolysosomal system (ES). Thus, maintaining the functional integrity of endolysosomes is crucial, which heavily relies on two key protein families: soluble hydrolases and endolysosomal membrane proteins. Particularly members of the TPC (two-pore channel) and TRPML (transient receptor potential mucolipins) families have emerged as essential regulators of ES function as a potential target in cancer therapy. Targeting TPCs and TRPMLs has demonstrated significant impact on multiple cancer hallmarks, including proliferation, growth, migration, and angiogenesis both *in vitro* and *in vivo*. Notably, endosomes and lysosomes also actively participate in various immune regulatory mechanisms, such as phagocytosis, antigen presentation, and the release of proinflammatory mediators. Yet, knowledge about the role of TPCs and TRPMLs in immunity is scarce. This prompts a discussion regarding the potential role of endolysosomal ion channels in aiding cancers to evade immune surveillance and destruction. Specifically, understanding the interplay between endolysosomal ion channels and cancer immunity becomes crucial. Our review aims to comprehensively explore the current knowledge surrounding the roles of TPCs and TRPMLs in immunity, whilst emphasizing the critical need to elucidate their specific contributions to cancer immunity by pointing out current research gaps that should be addressed.

## 1 Introduction

### 1.1 The ES in immunity

For a long time after the discovery of lysosomes in 1955 (1) they were branded as the recycling compartment of the cell. However, research has shown that their major functions go beyond degradation of cellular compartments. As part of the ES, they are key regulators in endocytic uptake, intracellular trafficking, signaling and regulation of membrane proteins (2, 3). Also, lysosomes have emerged as regulators specifically for calcium signaling, as they hold high intravesicular calcium concentration estimated to be around 500μM (4–6). In the context of immunity it has become evident that the ES plays a significant role in orchestrating immune responses (7, 8).

#### 1.1.1 Innate and adaptive immunity

In the innate immune system, the ES plays a pivotal role in recognizing pathogens through pattern recognition receptors (PRRs) expressed on immune cell surfaces. Activation of PRRs upon detection of pathogen-associated molecular patterns (PAMPs) triggers signaling cascades that activate immune cells and induce the production of proinflammatory cytokines and chemokines. Toll-like receptors (TLRs) located within endosomes and lysosomes, such as TLRs 3, 7, 8, and 9, specifically recognize pathogen-expressed nucleic acids and initiate kinase cascades to produce cytokines (9). The endolysosomal system’s ability to confine activation to foreign nucleic acids prevents recognition of self-DNA and RNA by degrading host nucleic acids using DNAse and RNAses (9, 10). Furthermore, the endolysosomal processing of TLRs is crucial for their activation, involving proteolytic cleavage by enzymes like AEP (asparagine endopeptidase) and cathepsins in various cell types (11).

In the adaptive immune system, endolysosomes are involved in the processing and presentation of antigens. The processing of antigens for presentation on MHC-II (major histocompatibility complex II) molecules involves proteases active in the endolysosomal pathway, facilitating their uptake from extracellular sources via icropinocytosis, phagocytosis or receptor-mediated endocytosis (12, 13). Within the endo-lysosomal compartments, the convergence of newly synthesized MHC-II molecules, proteolytic enzymes, and chaperones serves as a specialized environment for peptide loading, resembling a ‘reaction vessel’ for antigen processing (12, 13). While no specific endolysosomal protease is absolutely required *in vivo* (14, 15), efficient T cell activation can occur with lower antigen levels when captured by specific receptors like B cell membrane immunoglobulin (16). This mechanism involves early capture and stabilization of large antigen fragments, which can then be transferred to nearby MHC-II molecules. Moreover, the assembly of MHC-II/peptide complexes in late endocytic compartments prompts questions about their transfer to the cell surface, possibly achieved through fusion or vesicle transport (17). Notably, stimulation with TLR ligands or interaction with specific T cells induces tubulation of endolysosomes containing peptide/MHC complexes which offer an alternative mode of complex delivery (17, 18).

Interestingly, evidence suggests that the ES plays a significant role in the recycling and degradation of immune checkpoint molecules, with secretory lysosomes serving as temporary reservoirs for immune checkpoint proteins like cytotoxic T lymphocyte-associated antigen-4 (CTLA-4), programmed death-ligand 1 (PD-L1), TIM-3, CD70, CD200, and CD47 (19, 20). This degradation process might hinder the recognition of these immune checkpoint molecules by T cells, subsequently affecting the efficacy of T cell activation and immune checkpoint therapy.

#### 1.1.2 Tumor immunotherapy

Tumor immunotherapy represents an innovative approach leveraging the body’s immune system to identify and eradicate cancer cells, offering distinct advantages including high specificity, sustained efficacy, and minimal toxicity. This paradigm shift has emerged as a promising avenue in cancer treatment, evolving alongside conventional modalities like surgery, radiation therapy, and chemotherapy (21, 22). Notably, clinical immunotherapy methods encompass immune checkpoint inhibitors, adoptive cell therapy (ACT), and cancer vaccines, among others (23–25). Recent attention has turned to the pivotal role of lysosomes in cancer cell survival and the regulation of the tumor microenvironment (TME), positioning lysosomal targeting as a promising therapeutic avenue (26–28). For instance, Tang et al. introduced a pH-gated nano-adjuvant (PGN) designed to selectively modulate lysosomal pH and protease activity within tumor associated macrophages (TAMs), thereby promoting M1 polarization and enhancing antigen presentation (29). Additionally, lysosome degradation pathways have been explored for synergy with immune checkpoint inhibitors. Lysosome-targeting chimeric molecules (LYTACs) have demonstrated efficacy in degrading membrane proteins like epidermal growth factor receptor (EGFR) and PD-L1, bolstering therapeutic responses (26, 28). Also, lysosome trafficking modulation has been implicated in promoting the release of granular enzymes from tumor-infiltrating T cells (TILs) and activating innate immunity via toll-like receptors (TLRs), as demonstrated by enhanced immune responses observed with combination therapy involving cancer vaccines and TLR agonists (27, 30). Notably, the autophagy inhibitor chloroquine has shown promise in augmenting the efficacy of immune checkpoint inhibitors in clinical settings (31–33). CAR T cell therapy represents a form of cancer immunotherapy utilizing genetic manipulation to integrate chimeric antigen receptors (CARs) into the T cells of patients. While demonstrating significant efficacy in select cancer types (34), CAR T cell therapy encounters challenges concerning its persistence and functionality within the host organism, often resulting in tumor relapse or resistance (35). Lysosomes, specifically, exert a negative regulatory influence on CAR T cells by promoting the degradation of the CAR, thereby compromising CAR T cell activity and persistence. Recent research by Li et al. elucidated the underlying mechanisms, revealing that upon interaction with tumor antigens, the CAR undergoes ubiquitination and subsequent internalization, leading to lysosomal degradation and attenuation of CAR T cell function and persistence (36). To address this issue, a novel approach involving the development of a modified CAR, termed rCAR, was devised. This modified CAR features mutations in cytoplasmic lysine residues to reduce susceptibility to ubiquitination-induced downregulation. Notably, rCAR demonstrates the ability to resurface on the cell membrane following internalization, thereby maintaining functionality within intracellular compartments. In preclinical mouse tumor models, rCAR T cells exhibit elevated surface CAR expression, enhanced cytotoxicity, prolonged survival, and superior anti-tumor effects compared to conventional CAR T cells (36). In summary, lysosome-targeted cancer immunotherapy offers a multifaceted approach to disrupt tumor-immune interactions within the TME, potentially enhancing therapeutic outcomes. However, significant challenges including selective drug delivery, safety concerns, resistance mechanisms, and the need for elucidation of underlying mechanisms warrant further investigation.

This concise summary underscores the central role of the ES in both innate and adaptive immunity and in immunotherapy. Thus, proper function of endolysosomal compartments is crucial for proper regulation of immune response and surveillance. This connection is important to highlight, as impaired immune response and mechanisms that aid cancer cells to escape immune surveillance are prerequisites of cancer.

### 1.2 Impaired ion channel function in cancer cells

Impairment and manipulation of endolysosomal function have been shown to be one major mechanism of cells to sustain multiple cancer hallmarks (37). Therefore, one can say that proper lysosomal function is crucial for proper cell function. Lysosomal functionality is maintained by preserving low lysosomal pH and therefore securing enzymatic function through two main protein groups: the more than 60 hydrolytic enzymes located inside the lysosomes and lysosomal membrane proteins (2, 38, 39). The lysosomal membrane proteins and ion channels not only secure acidification of the lysosomal lumen, but also regulate protein imports from the cytosol, fusion with other organelles and transport of degradation products to the cytoplasm (40). The most prominent membrane proteins are the lysosome-associated membrane glycoproteins (LAMPs) and the vacuolar-type H^+^ ATPase (V-ATPase), an ATP-dependent proton pump. Past research has shown that V-ATPase does not only maintain the required low lysosomal pH (41), but it has become evident that it plays a significant role in cancer (42–45). Primarily, V-ATPase has been found to be overexpressed in cancer, which promotes ECM (extracellular matrix) degradation and consequently cancer cell invasion and migration (46). Additionally, inhibition of V-ATPase has been shown to reduce cancer cell proliferation, induce apoptosis and promote mitochondrial fission (43, 47). The unravelling of the role of V-ATPase and hence lysosomal ion homeostasis in cancer progression was followed by multiple clinical trials. However, V-ATPase inhibitors largely failed in clinical trials (48), which shifted the limelight to further lysosomal ion regulators, i.e. ion channels and their potential role in maintaining cancer hallmarks. The focus of this research field centers around two-pore channels (TPCs) and transient receptor potential cation channels (TRPMLs) both of which are cation channels abundantly co-expressed in the lysosomal membrane of non-carcinogenic and carcinogenic tissue (2, 49).

#### 1.2.1 TPCs

TPCs are ligand gated cation ion channels embedded in the membranes of endosomes, late endolysosomes, and lysosomes (50). In the human genome TPC1 and TPC2 are the two known paralogs, TPC1 is mainly found in endolysosomal organelles, whilst TPC2 is predominantly expressed in lysosomes [21]. Structurally, TPC2 is composed of two homologous building blocks, each consisting of six transmembrane domains with N- and C-termini reaching into the cytosol (51). Nicotinic acid adenine dinucleotide phosphate (NAADP)- and PI (3,5)P2 are both endogenous agonists of TPC2, which trigger the release of different cations in an agonist-depending manner (52–55). What has been recognized so far, is a well-established model of NAADP indirectly activating TPC2, resulting in a release of calcium from acidic stores (52, 55). Also, it has been proposed recently that TPC2 can function as sodium permeable ion channel upon direct activation by PI (3,5)P2 (54, 55). It has been shown that TPC2 plays a crucial role in tumorigenesis, as amplifications in the TPC2 gene and overexpression of the channel are associated with cancer hallmarks. Müller et al. demonstrated that TPC2 drives cancer cell proliferation and is crucial for sustaining cellular energy metabolism (56). Also, the genetic ablation of TPC2 resulted in a significant reduction of cancer cell proliferation *in vitro* (56–58) and prevented tumor growth *in vivo* (56). Besides driving proliferation, TPC2 is an activator of migration and invasion and therefore is involved in two critical steps in the development of metastases (57, 59). It has been indicated that silencing TPC2 leads to a minimization of lung metastases *in vivo* and reduced cell migration by impairment of the β1-integrin recycling pathway (57). Moreover, TPC2 is involved in promoting angiogenesis by being part of the VEGFR2/NAADP/TPC2/Ca^2+^ signaling pathway, which promotes VEGF-induced angiogenesis *in vitro* and *in vivo* (60, 61).

#### 1.2.2 TRPMLs

TRPML is a subfamily of the transient receptor potential (trp) family are non-selective cation channels, which are also ubiquitously expressed in late endosomes and lysosomes of mammalian cells (62). There are three known mammalian isoforms of TRPML: TRPML1, TRPML2 and TRPML3, which were established upon the identification of TRPML1 as the protein mutated in the lysosomal storage disease mucolipidosis type IV (MLIV) (63). PI (3,5)P2 and reactive oxygen species (ROS) act as endogenous activators and trigger lysosomal calcium release from TRPML1 in a pH-dependent manner, whereas PI (4, 5)P2 represents the endogenous inhibitor at membrane sites (64, 65). In addition to being considered the primary lysosomal calcium channel, TRPML1 also demonstrates the ability to transport heavy metals such as Fe^2+^ and Zn^2+^ from the lysosomal lumen into the cytosol (66). It has been suggested that the release of calcium through TRPML1 drives fission and fusion within the endocytic pathway and lysosomal membrane trafficking as many trafficking steps are calcium-dependent and altered in knockout cells (66–68). Likewise, the importance of TRPML1 in cell signal transduction, organelle homeostasis and endosomal maturation have been demonstrated (67, 69–71). In cancer, a study by Abrahamian et al. revealed elevated levels of TRPML1 in different cancer cell lines (72). Further research has revealed a significant impact of TRPML1 on sustaining multiple cancer hallmarks such as cell proliferation, survival, invasion and migration (73–77). Also, it has been recognized that TRPML2 plays an important role in cancer development and therapy. The expression of TRPML2 in cancer tissue has been connected to higher proliferation rates and impaired apoptosis, whilst overall survival of patients negatively correlates with TRPML2 expression levels (78–80).

**Figure 1:**
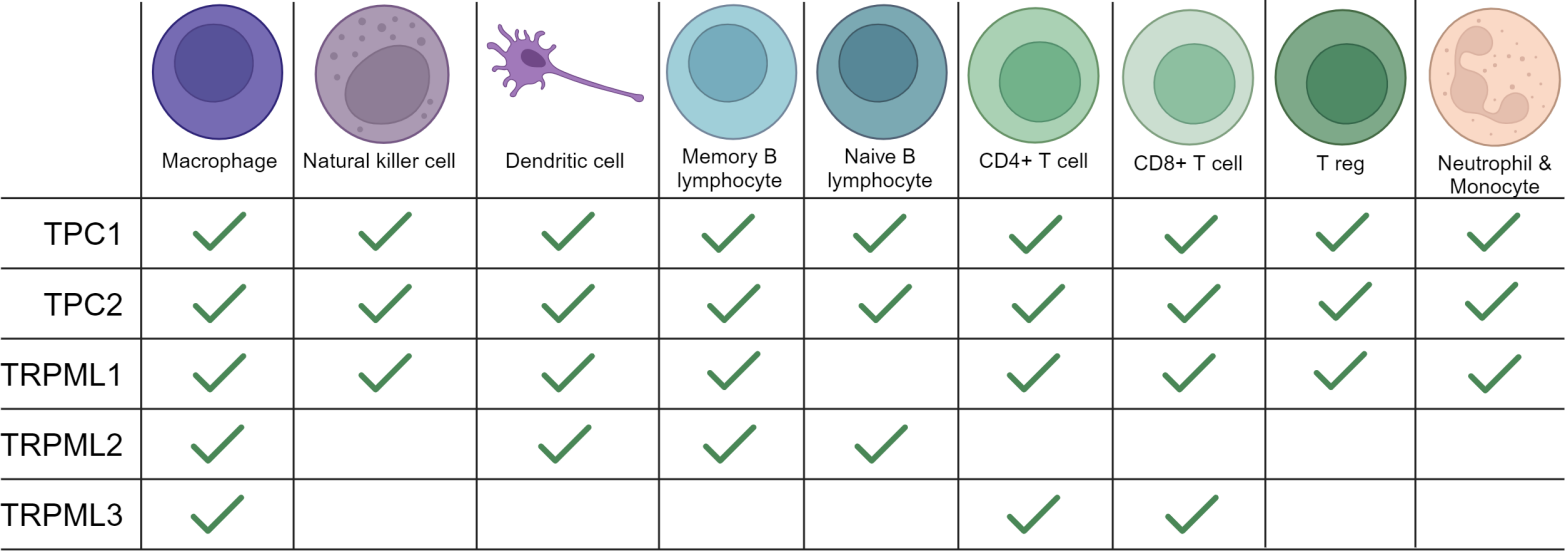
Expression of TRPMLs and TPCs in immune cells. An overview of the expression of TRPMLs and TPCs among immune cells according to the literature used in our review and additional data from Human Protein Atlas: Blood Cell Type Expression (RNA) of TPCN1/TPCN2/MCOLN1/MCOLN2/MCOLN3 available at https://www.proteinatlas.org.

Taken together, emerging research has highlighted the key role of two lysosomal membrane families TPCs and TRPMLs in cancer development and tumorigenesis by sustaining several cancer hallmarks, e.g. cell proliferation, evading apoptosis, chemoresistance and migration of invasive cancer cells (56, 57, 81, 82). The involvement of the ES and its ion channels in endocytosis, exocytosis, and trafficking processes in cancer cells, as well as the involvement of the ES in regulating major trafficking processes in immune cells prompts an intriguing query, which has not been addressed yet: Are TRPMLs and TPCs also regulating exocytosis processes in immune cells and therefore, responsible for cytokine release? As both ion channel families are expressed in different immune cells (**Figure 1**) they might also regulate immune cell function. Also, does impaired function of TRPMLs and TPCs in immune cells lead to altered expression of i.e.: MHC molecules? Are both ion channel families key regulators of the mechanisms we observe in cancer cells and immune cells, that aid to evade immune destruction and surveillance? In this review we want to summarize the current knowledge of both ion channel families in immune cells to then discuss their potential role in cancer immunity and to identify possible research gaps that should be addressed in the future.

## 2 The role of TRPMLs in immunity

**Figure 2:**
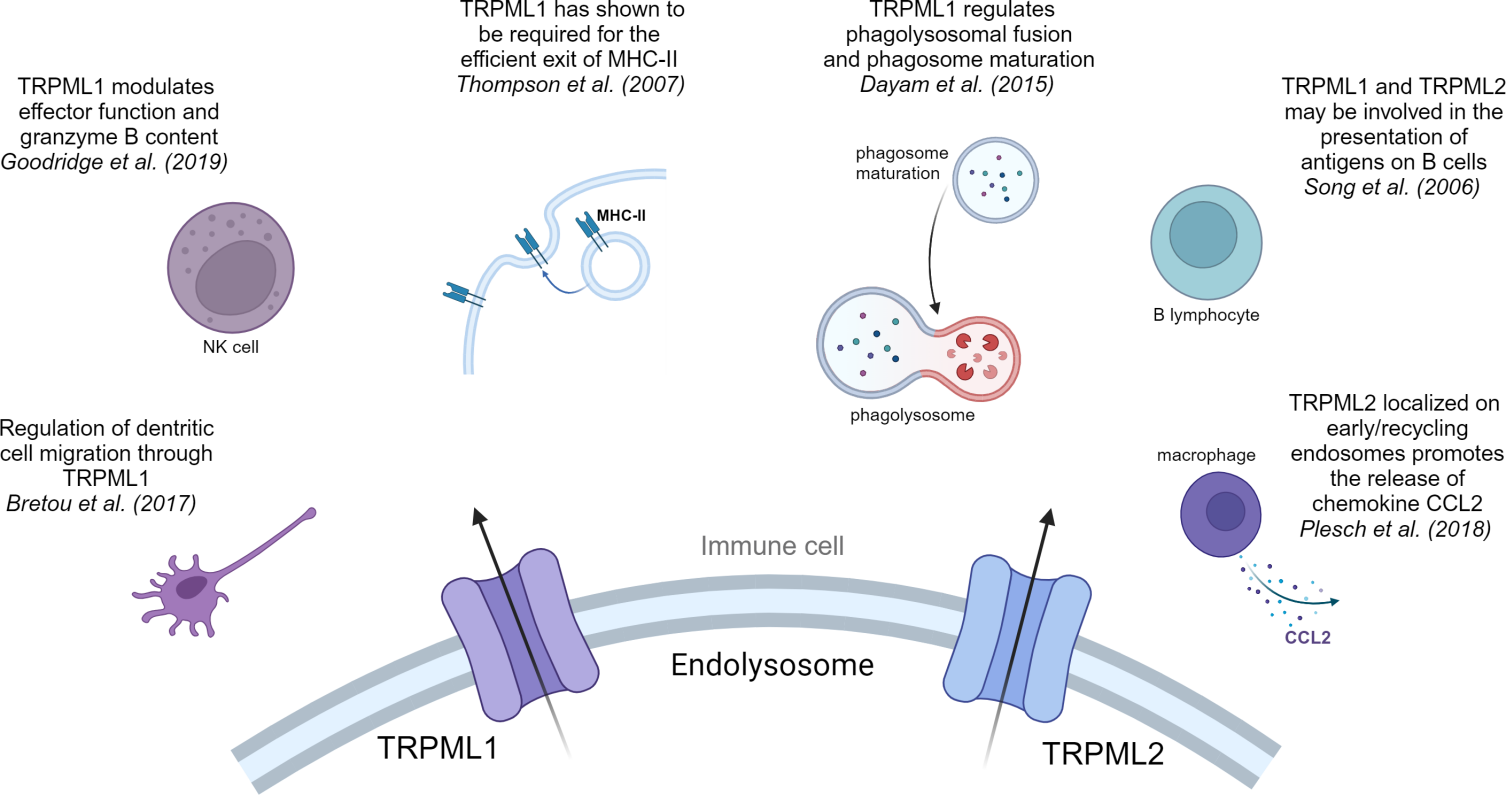
TRPML1 and TRPML2 in immune cells. Overview of the pivotal effects that cation channels of the TRPML family have in immune cells.

Mucolipins have been shown to play a pivotal role in the autophagic regulation, calcium homeostasis and cancer metastasis (81, 83, 84). Besides their role in cancer, recent research highlights their striving importance in immunity. In immune cells two of the three known isoforms, TRPML1 and TRPML2, are mainly expressed in monocytes and macrophages, whereas expression of TRPML3 is rather low (85). Thus, we will next focus on what is known about TRPML1 and TRPML2 in immune cell function (**Figures 1**, **2**).

### 2.1 Macrophages

Macrophages play an important role within the innate immune system by secreting chemokines and cytokines after stimulation and thereby attracting more immune cells to the inflammation herd, as has been excellently reviewed elsewhere (86). The polarization state of macrophages is determined by the presence of different cytokines. On the one hand, classically activated macrophages (M1) are polarized through interferon-γ, TLR-ligands, or microbial substrates (e.g., LPS). The activation to M1 is linked to an increase in the production of pro-inflammatory cytokines such as TNF-α, IL-6, IL-1, and IL-23, consequently leading to the production of reactive oxygen species (ROS). On the other hand, M2 macrophages are generated in the presence of IL-4 and IL-13, and play a role in suppression of inflammation and tumor progression through the secretion of anti-inflammatory cytokines such as IL-10 and TGF-β (86, 87).

One of the main functions of macrophages is phagocytosis, a special form of endocytosis and a crucial step in the defense against pathogens and tumor cells (88). Recent work showed that TRPML1 plays different roles in the process of phagocytosis. Samie et al. were the first to provide information of the key role of TRPML1 in phagocytosis (89). They have claimed the involvement of TRPML1 in the process of engulfment of large particles by enlarging the cell surface/membrane through lysosomal exocytosis (89). They suggested that particle binding stimulated the endolysosomal kinase PIKfyve, which leads to a production of PI (3,5)P_2_ through phosphorylation of PI (3)P. PI (3,5)P_2_ is an endogenous activator of TRPML1 (90), thus the increased intracellular PI (3,5)P_2_ levels activate TPRML1, triggering a Ca^2+^ release which in return induced lysosomal exocytosis at the site of the phagocytic cup (89). In line with their observations, they demonstrated that a knockout of TRPML1 decreases the phagocytosis of large particles in bone marrow derived macrophages (BMDM), hinting that the TRPML1-mediated Ca^2+^ release is crucial for large particle phagocytosis (89). Moreover, Dayam et al. demonstrated that phagolysosomal biogenesis was impaired in TRPML1 silenced or PIKfyve inhibited cells and halted phagosome maturation (91). They indicate that TRPML1 regulates phagolysosomal fusion and phagosome maturation via PIKfyve-TRPML1-Ca^2+^ axis (91, 92), consequently leading to enhanced nuclear translocation of TFEB (transcription factor EB) (93). This observation suggests that nuclear translocation of lysosomal master regulator TFEB is dependent on phagosome maturation (93).

Besides phagocytosis, cytokine secretion by macrophages is a crucial step in the process of immune cell growth and activation. In this context, the treatment of macrophages with LPS triggers their activation followed by a strong upregulation of TRPML2 expression, while TRPML1 and TRPML3 expression remained unchanged (94). Plesch et al. followed up on this observation and showed that TRPML2 is localized on tubular compartments and early/recycling endosomes, where it promotes the release of major chemokine CCL2 (95). Subsequently, the release of CCL2 from macrophages results in a recruitment of more macrophages. In the TME release of cytokines like CCL2 and other growth factors from a network of different immune cells significantly contributes to tumor progression (96).

Moreover, alongside dendritic cells (DCs) and B-lymphocytes, macrophages are professional antigen presenting cells (APCs). Therefore, the presentation of exogenous antigens through MHC-I or MHC-II molecules counts as another major function of macrophages. Antigen presentation via MHC molecules builds a bridge between innate and adaptive immune system and is crucial for an effective immune response and surveillance. In retrospect TRPML1 was required for the efficient exit of MHC-II and MHC-I molecules that target the plasma membrane in macrophages (97).

### 2.2 Dendritic cells and natural killer cells

DCs serve as crucial APCs in the adaptive immune response (98, 99). After capturing pathogenic antigens through macropinocytosis, immature DCs undergo maturation. Subsequently, they process and exhibit elevated levels of antigens, stimulatory molecules, and cytokines. Upon migration to lymph nodes, mature DCs present these antigens and activate T cell responses (100, 101). Bretou et al. have highlighted the importance of TRPML1 in DC chemotaxis and migration to lymph node by controlling the motor protein myosin II retrograde flow at the cell rear (102). Moreover, they demonstrated that TRPML1-mediated Ca^2+^ efflux promotes TFEB translocation in DCs. Translocation of TFEB results in an activation of the CLEAR network, which has been linked to a downregulation of MHC-I expression and an upregulation of MHC-II expression, IL-6, TNF-alpha and IL-1β production and most importantly DC migration (10, 102–105). Now, TRPML1-mediated TFEB translocation is responsible for reduced MHC-I expression on DCs. This prompts the question whether a TRPML1-dependent reduction is also observable on cancer cell surface and therefore accountable for a reduced detection by CD8^+^ T cells.

In addition, there is evidence that TRPML1 is a key regulator in the response of cells to ssRNA via TLR7 pathway (106). In this case, Li et al. have shown that by inhibiting PIKfyve/TRPML1 function the transport of ssRNA into the lysosomes is altered. An impairment of ssRNA transport, leads to an impaired TLR7 response, which is followed by reduced maturation/activation of dendritic cells and impeding their migration to the lymph nodes. Yet again, this highlights the important role of PIKfyve/TRPML1 axis in immunity (106).

The main functions of nature killer (NK) cells are to limit the spread of microbial infections and tumors and trigger “induced self-killing” via receptor binding (107). Conversely, in this class of immune cells TRPML1 plays a modulating role, regulating effector function and granzyme B content. A siRNA knockdown of TRPML1 or inhibition of upstream regulator PIKfyve led to an enlargement of lysosomes with increased granzyme B content (108). Therefore, Goodridge et al. suggested that by manipulating lysosomal calcium homeostasis, e.g.: targeting TRPML1 in NK cells, one can increase NK cell function and efficacy (108).

### 2.3 B lymphocytes

B lymphocytes constitute an integral component of the adaptive immune system, synergizing with other immune effectors such as T cells, macrophages, and DCs to eliminate foreign antigens. Their function involves the production and secretion of numerous antibody molecules, enabling them to recognize and respond to pathogens or foreign antigens (109, 110). Central to this process is the B cell receptor (BCR), an integral membrane protein characterized by its structural composition of two Ig light chains, two Ig heavy chains, and one heterodimer composed of Igα and Igβ. Notably, B cells harbor a specialized lysosomal compartment where antigens originating from endocytosed BCRs are loaded onto MHC-II molecules (109).

TRPML1 and TRPML2 have been shown to be expressed in B cells (111). Song et al. showed that TRPML1 deficient B cells do not show gross changes within the lysosomal compartments (112). This is in line with their observation that patients with mucolipidosis type IV (MLIV), who genetically lack TRPML1, do not show abnormalities in lymphocyte function or any immune function defects. Song et al. suggested compensatory lysosomal mechanisms by TRPML2 or other ion channels to be responsible for the maintenance of immune function upon TRPML1 ablation (111, 112).

Taken together there is growing evidence that TRPML1 and TRPML2 are essential for the rapid response of the innate immune system and are also key mediators between innate and adaptive immune response. There lays great potential in further elucidating their precise role in adaptive immunity in general, but also in elucidating their role in cancer immunity. The discussed data suggests that TRPML1 and TRPML2 are important regulators of phagocytosis, phagosome biogenesis, immune cell movement and are involved in mediating cytokine release. All named biological processes where both ion channels play key roles are also found in mechanisms that help cancer cells evade immune destruction. Thus, it would be relevant to know whether TRPML1 and TRPML2 play a role in the release of other cytokines important for tumor growth and securing a pro-tumorigenic TME. If TRPML1 affects MHC-I and II expression on macrophages, does it also regulate MHC-I expression in cancer cells and therefore cytotoxic T cell response? As TRPML1 affects cancer cell as well as DC migration and the recycling of adhesion molecules as well as actin remodeling, is leukocyte recruitment impacted by TRPML1? Could targeting TRPML1 ion channels in cancer present a dual role, by inhibiting cancer hallmarks on the one hand and fostering an anti-tumor response on the other?

## 3 TPCs in immunity

**Figure 3:**
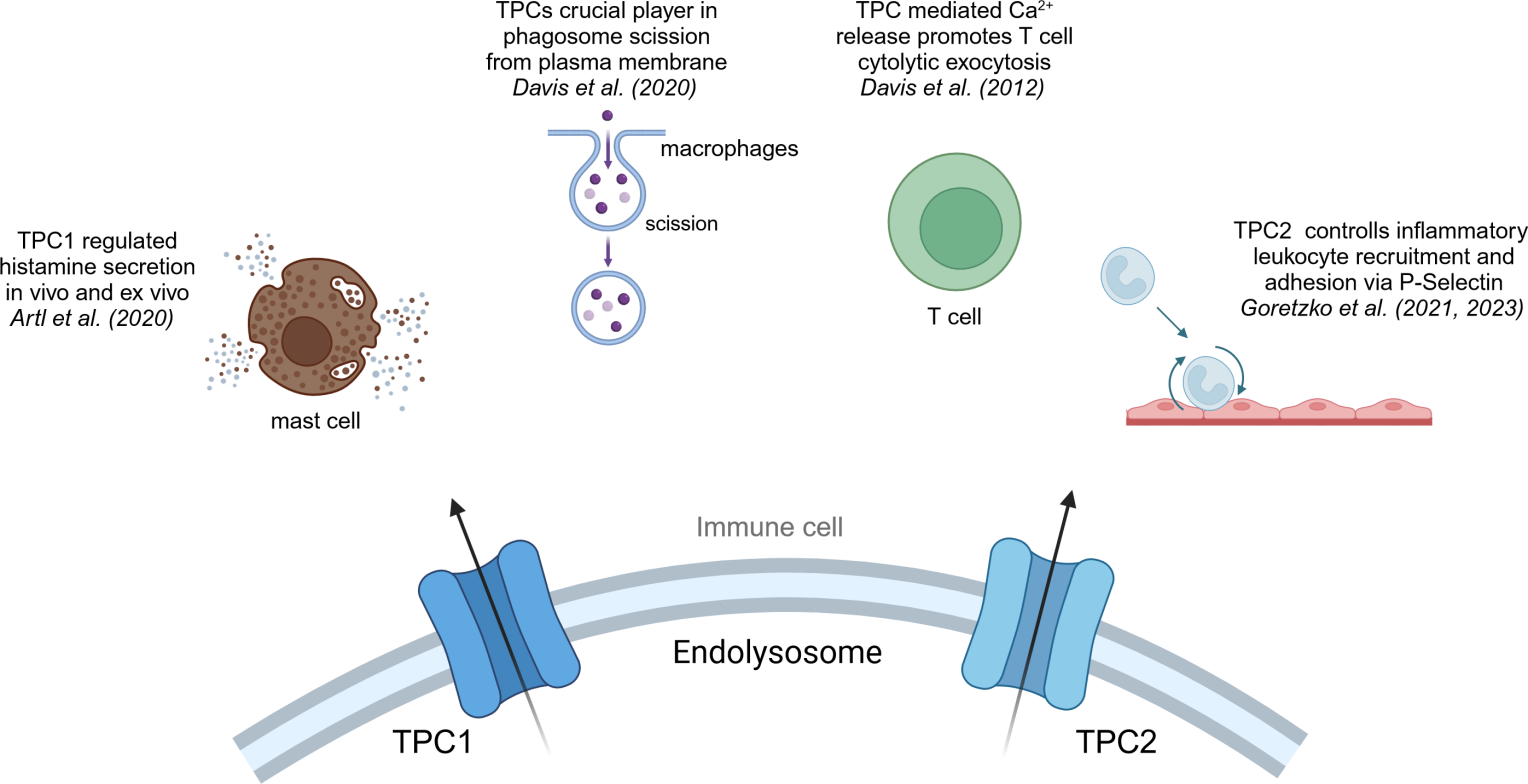
TPC1 and TPC2 in immune cells. Overview of the effects that cation channels of the TPC family have in immune cells.

Both isoforms of the TPC family, TPC1 and TPC2, have been reported to be involved in calcium homeostasis, cell regulation, cancer and the regulation of immune responses (38, 56) (**Figure 3**). TPC1 as well as TPC2 are expressed in all immune cells, yet they show higher expression levels in monocytes and neutrophils compared to other immune cells (113).

### 3.1 Macrophages

As mentioned, phagocytosis is a crucial step in macrophages and part of the first line defense of the innate immune system against pathogens. A recent study by Davis et al. suggested that TPC1 or TPC2 may be a crucial player in phagosome scission from plasma membrane, a key step in the process of phagocytosis (114). They imply that Fcγ receptor binding triggers the release of local lysosomal Ca^2+^ via NAADP/TPC pathway, which in return activates the GTPase dynamin 2, an essential protein for phagosome scission from the plasma membrane (114). Moreover, Li et al. could show that a blockage of TPC2 in BMDMs resulted in reduced activation of the immune system, due to endolysosomal trapping. They have shown that TPC2 deficient cells are characterized by a reduced IL-1β levels and therefore diminished immune activation (115). Yet, further investigations in regard to the effect on cancer cells and the TME have not been made. Another question to be answered is whether TRPML or TPC channels are predominantly regulating macrophage function, or if both are mandatory for proper cell function.

Another mode of action has been described by Goretzko et al. emphasizing the role of TPC2 as a key element in controlling inflammatory leukocyte recruitment and adhesion via P-Selectin (116, 117). P-Selectin is a transmembrane protein that plays a crucial role in the initial recruitment of leukocytes to the site of inflammation. In the context of cancer immunity, P-Selectin has been linked to the development of cancer metastasis by complex forming with tumor cells and leukocytes, and thereby masking tumor cells from recognition by macrophages (118). Also, E-Selectin, which shows similar functional roles in leukocyte recruitment and cancer metastasis like P-Selectin, appears to be highly co-express with TPC2 in hepatocytes and in that way may promote an inflammatory TME (119, 120).

### 3.2 Mast cells

TPC1 has been linked to the exocytosis event of histamine secretion in mast cells (121). Histamine secretion is an inflammatory signal by mast cells and basophils and marks the main driver of allergic reaction and is dependent on intracellular calcium signals (122). Arlt et al. suggested TPC1-mediated Ca^2+^ release to be regulating histamine secretion *in vivo* and *ex vivo* in murine mast cells. The activation of Fcε receptors by IgE antibodies induces the activation of IP3 receptors, leading to the release of Ca^2+^ from the endoplasmic reticulum (ER) (123). The consequent elevation in cytosolic free Ca^2+^ concentration initiates the exocytosis of secretory granules. It was hypothesized that TPC1 predominantly resides at the interface between endolysosomes and the ER, facilitating the absorption of Ca^2+^ from the ER. It has been shown that TPCs play a role in the regulation of innate immune system and are invaluable during anaphylaxis (121). Thus, it may be important to shed more light on the presentation of chemokine signaling molecules and cytolytic factors in cytotoxic T cells, as ideally, a cytotoxic T cell response against tumor tissue is the aim of tumor immune therapy approaches.

### 3.3 T cells

Cytotoxic CD8^+^ T cells (CTLs) are indispensable in immune defense by targeting and eliminating infected cells, as well as malignant cells. They achieve their killing function by recognizing foreign or mutated peptides presented on the cell surface through MHC-I molecules. By secreting granzymes and perforin, they induce apoptosis and effectively eliminate their target cells. Indeed, the activation of TPCs in CTLs initiates the extracellular release of perforin and granzyme B from cytolytic granules. Notably, studies have identified the presence of both TPC1 and TPC2 within the immunological synapse, the interface between CTLs and target cells (124). However, the specific implications of TPC activation on anti-tumor responses have yet to be thoroughly investigated (125). In CD4^+^ T cells the role of TPCs is not fully understood and elucidated. Dammermann and Guse et al. have not seen a significant effect of NAADP mediated endolysosomal Ca^2+^ release in human Jurkat T cells (126). However, the importance of long lasting Ca^2+^ signaling for T cell activation is well elucidated (127–129). Thus, TPCs might be involved in the regulation of Ca^2+^ signals in CD4^+^ in the same manner as described in mast cells by Arlt et al. (121).

It becomes clear that research on the role of TPCs in immunity is emerging and it is of vast interest to address research gaps. TPCs are abundant for lysosomal trafficking in cancer cells as well as in immune cells, thus investigating their mechanisms and precise roles remains of great significance. Multiple queries remain elusive: Do TPCs regulate cytokine release in macrophages and cancer cells as observed for TPC1 in mediating histamine secretion in mast cells? Is the enhancement of CTL-mediated antitumor responses mediated by TPCs? Since there are no evidences for TPCs in the recycling of MHC molecules and consequently MHC-I and MHC-II antigen representation, we could wonder whether TPCs play a role in the process of antigen presentation.

## 4 Discussion

### 4.1 Potential role of TRPMLs and TPCs in cancer immunity

**Figure 4:**
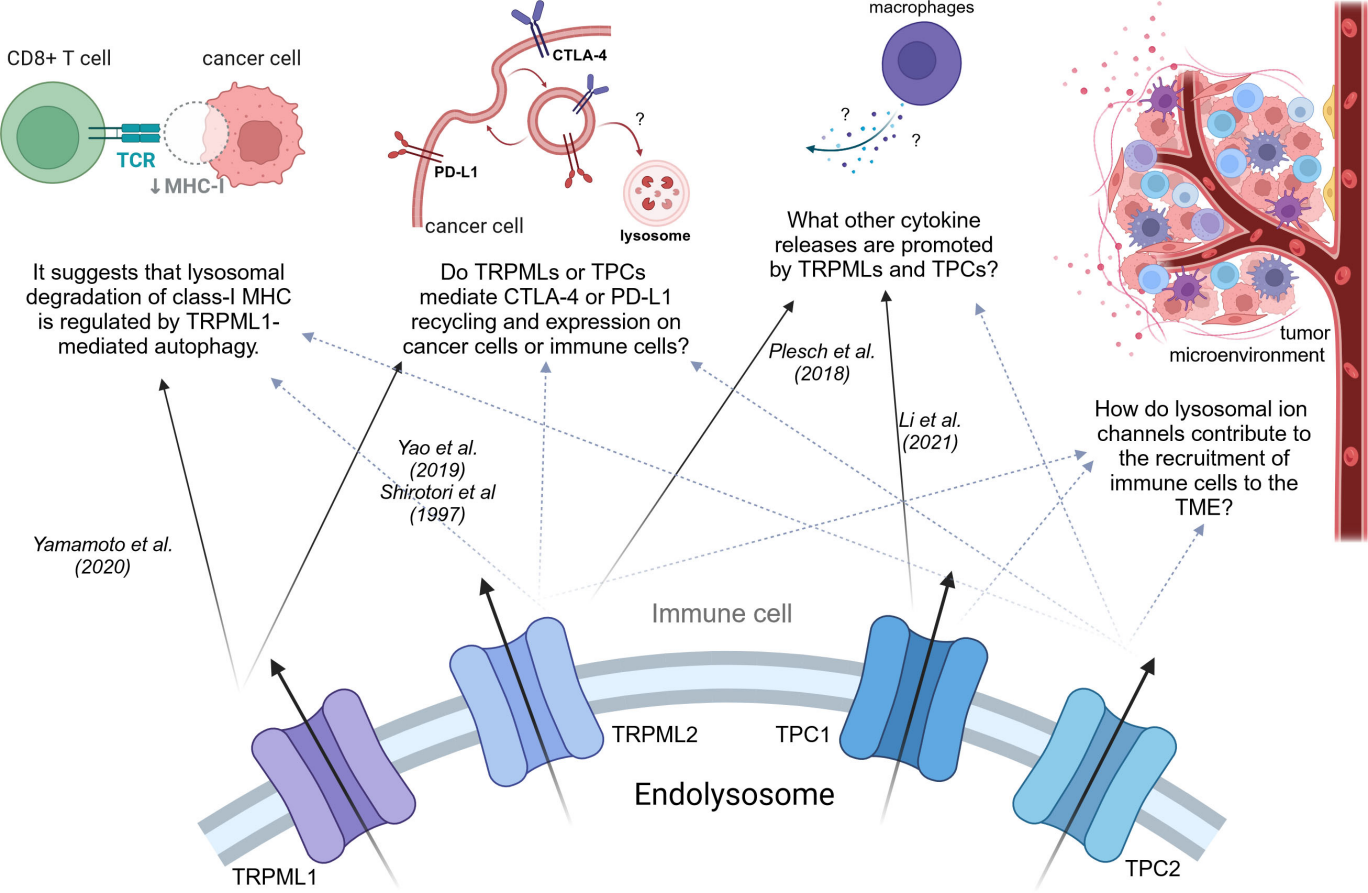
Potential effects of TRPMLs and TPCs on cancer immunity. Effects that might be governed by TRPMLs or TPCs and therefore aid cancers to evade immune surveillance or destruction.

The information presented here sheds light on the roles of TPCs and TRPMLs in cancer and immunity. Yet, a significant challenge persists in connecting these roles to elucidate their impact on cancer immunity, particularly in aiding cancer cells to evade immune destruction. To explore this, our discussion will be divided into two main sections: mechanisms contributing to the stabilization of the TME and mechanisms facilitating evasion of immune destruction (**Figure 4**).

### 4.2 The TME

Tumor progression is very closely connected and dependent of the contribution of the environment surrounding the tumor. Thus, the concept of a TME has been studied extensively over the past century, elucidating that it is shaped and trained by cancer cells to assist the development of cancer hallmarks. The TME can be seen as a complex inflammatory system surrounding tumors, among others consisting of fibroblasts, endothelial cells, extracellular matrix, blood vessels, but also immune cells, signaling molecules and metabolites (130, 131). That immune cells are part of the TME has been known, since tumor-infiltrating lymphocytes (TILs) were identified (132). Ever since then, investigating and characterizing immune cells in the TME has been a high priority. Due to their altered characteristics, immune cells in the TME are regarded to serve as a potential target for cancer immunotherapy (133).

Recruiting immune cells to the TME and sustaining a pro-tumorigenic and inflammatory setting heavily relies on exocytosis processes such as cytokine secretion (131, 134). Specially TRPMLs have been linked to be key regulators of lysosomal exocytosis in healthy and malignant tissue (74, 89, 104, 135). In this context, the influence of TRPML1 on lysosomal exocytosis in healthy tissue was nicely described by Medina et al. They demonstrated that TFEB controls lysosomal exocytosis at the transcriptional level (136, 137) by promoting an increase in intracellular calcium levels via TRPML1 (138). Additionally, in Parkison Disease (PD) activation of TRPML1 rescued the impaired secretion of α-synuclein (α-syn) and prevented its accumulation within cells. These findings indicate that the levels of α-syn inside cells are influenced by lysosomal exocytosis in human dopaminergic neurons (139). This insight suggests that targeting TRPML1, and thereby lysosomal exocytosis, could offer a promising therapeutic strategy for PD and other disorders characterized by impaired lysosomal exocytosis. In macrophages, Plesch et al. demonstrated that TRPML2 governs release of chemokines, such as CCL2 (95). Further elucidation on this regard has not yet been made. However, the CCL2-CCR2 axis is one of the major chemokine signaling pathways (96), which is not only involved in tumor progression, but also helps to create an inflammatory TME. The TRPML2-mediated release of CCL2 leads to a M2-type polarization of macrophages in the TME. Furthermore, M2 macrophages in the TME or even TAMs, which are thought to have M2-like features, secrete CCL2 themselves to educate a larger number of macrophages to the TME, thus increasing their number of associates (111). In conclusion, the TRPML2/CCL2 axis might contribute to a pro-tumorigenic TME by recruiting and repolarization of immune cells and therefore in securing tumor growth (140).

To conclude, exocytosis is known to regulate TME adaptation, such as acidification and cytokine release, which shape vascularization and immune response (130, 131). TRPMLs have shown to be responsible for exocytosis processes and secretion of cytokines (95, 135). TPC1 is proposed to be involved in exocytosis behavior in mast cells (121), but no data in cancer is known so far. Still, there is a vast research gap on the effect of TPC and TRPML mediated lysosomal exocytosis on TME formation and maintenance. Thus, it would be beneficial to investigate, if and how, other lysosomal ion channels contribute to cytokine secretion and consequently the recruitment of immune cells to the TME? What cytokines/growth factors might be released through TPC or TRPML-mediated exocytosis and what effect does the release have on a pro-tumorigenic environment?

### 4.3 The lysosome and tumor-promoting inflammation

The lysosome plays a critical role in driving inflammatory responses that can facilitate proliferation, DNA damage, and angiogenesis (141). Lysosomal enzyme release can initiate inflammation in various contexts. For instance, cathepsin B-mediated cleavage of trypsinogen-1 in the pancreas can induce pancreatitis (142), a chronic inflammatory condition associated with heightened pancreatic cancer risk (143). Deficiencies in essential autophagy genes like Autophagy related 7 (ATG7) and Beclin 1 can also trigger chronic inflammation across multiple settings (144) and are implicated in the promotion of spontaneous cancers in organs such as the lung, liver, and lymphocytes through mechanisms that are not fully elucidated (145). Additionally, therapy-induced lysosomal membrane permeabilization may contribute to inflammation preceding cell death (146).

Heparin sulfate proteoglycans (HSPGs), a major component of the extracellular matrix, modulate the activity of cytokines and growth factors such as TGF-β (147). Additionally, HSPGs can induce inflammatory responses through TLR 4 signaling activation and leukocyte recruitment (148). Heparanase, the sole known mammalian endoglycosidase capable of degrading HSPGs (149), is synthesized as an inactive proenzyme and undergoes proteolytic activation in the lysosome. Inhibitors of heparanase, including heparin sulfate mimetics like PG545, PI-88, M402, and SST0001, are under investigation in clinical trials and have demonstrated promising efficacy in impeding progression and metastasis in ovarian cancer and hepatocellular carcinoma (150, 151).

Furthermore, the lysosome plays a crucial role in regulating glucocorticoid levels and anti-inflammatory responses in diseases like lupus erythematosus and rheumatoid arthritis (152). Cancer cells often display elevated expression of the glucocorticoid receptor (153–156), and inhibition of lysosomal function with compounds like chloroquine or the V-ATPase inhibitor bafilomycin A1 can mitigate inflammation. Besides lysosomal pump V-ATPase, do TPCs or TRPMLs affect the expression of glucocorticoid receptors? Conversely, knockdown of TFEB leads to increased glucocorticoid levels and suppression of proinflammatory cytokine transcription (152). In this context, TRPML1-mediated calcium efflux has been connected to mediate TFEB translocation to the nucleus (135, 157). Thus, the question arises, whether TRPML1 activation and/or overexpression in cancer cells, result in enhanced transcription of proinflammatory cytokines. Does targeting TRPML1 in cancer result in a reduced release of proinflammatory cytokines?

Inflammation mediated by heparanase, cathepsins and their substrates, or modulation of the glucocorticoid receptor may impact distinct tumor initiation pathways triggered by chronic inflammation. However, whether and how lysosomal ion channels are involved in tumor-promoting inflammation needs further investigation.

### 4.4 Evading immune destruction

During cancer therapy, it has been noted that autophagy coupled to lysosomal degradation is a key mechanism of resistance to immunotherapy. In pancreatic cancer cells, it has been demonstrated that autophagic processes are linked to lysosomal degradation of MHC-I (33). Consequently, reducing the expression of MHC-I at the cancer cell surface and the recognition by CD8^+^ T cells, hampering the efficacy of immunotherapy. Interestingly, TRPML1 has been linked to regulate autophagic processes in cancer cells (64, 84, 158, 159). In the context of tumor cell survival within a complex environment, TRPML1 activation has been shown to promote autophagy in several cancer cell lines by disrupting the fusion process between autophagosomes and lysosomes. Rosato et al. demonstrated that TRPML1 activation leads to the formation of autophagic vacuoles (AVs) independently of TFEB, indicating TRPML1’s ability to influence autophagy through two distinct mechanisms: rapidly, by promoting both AV formation and fusion with lysosomes, and persistently, by facilitating TFEB nuclear translocation and subsequent transcription of genes essential for autophagy and lysosomal function (160), subsequently ensuring a sustained supply of autophagic substrates during prolonged autophagy stimulation. Furthermore, this process engages a feedback loop wherein TFEB positively regulates TRPML1 mRNA expression, thereby reinforcing long-term autophagic activity (84, 160). Moreover, it has been shown that the inhibition of autophagy induced by TRPML1 leads to disruptions in mitochondrial turnover and mitophagy. This disruption subsequently triggers an increase in ROS, causing significant DNA damage within cancer cells (64, 158) and activating ROS sensor TRPML1 (64). Therefore, it suggests that lysosomal degradation of MHC-I observed in pancreatic cancer is regulated by TRPML1-mediated autophagy.

Recently, immune checkpoint therapy has been proven as a breakthrough in cancer therapy (161). While many immune checkpoints have been identified in the past decades, only two are being directly targeted in cancer immunotherapy so far: CTLA-4 and PD-1 and its respective ligand PD-L1 (162, 163). CTLA-4 is an inhibitory checkpoint molecule, mainly located at the plasma membrane of T cells (164). Following T cell receptor (TCR) activation, CTLA-4 undergoes upregulation and binds to B7 with greater avidity than the T lymphocyte receptor CD28. This interaction leads to decreased T cell proliferation and reduced cytokine secretion (165–168). In the initial stages of tumorigenesis, CTLA-4 can reduce T cell activation by generating inhibitory signals, thereby attenuating the immune response against the tumor (169). Recently, research has shown that cancer cells express CTLA-4 in their favor. The binding of T cells to CTLA-4 expressed by cancer cells leads to a reduced T cell activation towards the cancer and subsequently reduced overall anti-tumor response (170–172). Lysosome have shown to play a central role in recycling and degrading CTLA-4. It is known that CTLA-4 can be internalized via endocytosis (173, 174) and subsequently degraded in the lysosome or bind to activator protein 1 (AP1) and AP2 to be degraded (175, 176). However, how recycling processes of CTLA-4 to the plasma membrane has not been highlighted yet.

Programmed cell death protein-1 (PD-1) serves as a prominent immunosuppressive checkpoint, primarily expressed in macrophages, B lymphocytes, dendritic cells (DCs), monocytes, tumor-specific activated T cells, myeloid cells, and natural killer (NK) cells during prolonged exposure to antigens (177, 178). One of the ligands for PD-1 is PD-L1, which is predominantly expressed in tumor cells, tumor-infiltrating cells, and APCs across various cancer types (177). In general, T cell activation predominantly hinges on dual signals. The initial signal involves the binding of MHC-presented antigens to the TCR. The second signal comprises co-stimulatory and co-inhibitory signals (179). The engagement between PD-1 on T cells and PD-L1 on tumor cells or APCs can actively impede T cell activation. This interaction is known to lead to T cell apoptosis, reduced cytokine production, T cell lysis, and the induction of antigen tolerance, enabling the tumor to evade immune surveillance (180, 181). PD-L1 is not solely found on the cell surface; it is also found intracellularly and its expression levels are dynamically regulated by lysosomal-related dynamic transport and degradation mechanisms (182–184). Moreover, patients treated with approved immune checkpoint inhibitors or nivolumab (mAb against PD-L1) (185) or ipilimumab (mAb against CTLA-4) (186), have been shown to develop resistance during therapy. Remarkably, poor outcomes of immunotherapy has been connected to lysosomal degradation of immune checkpoint PD-L1, yet specific mechanisms of resistance of immune therapy remains elusive (185, 187).

In summary, both immune checkpoints CTLA-4 and PD-L1 are expressed on cancer cells, contributing to immune evasion. Their expression on the cell membrane, as well as their recycling, has been shown to be regulated through the ES in which TRPML1 or TPC2 might be key players. Do TRPMLs or TPCs influence CTLA-4 or PD-L1 recycling and expression on cancer cells or immune cells? Are impaired TPCs or TRPMLs function responsible for the observed resistance mechanisms during immunotherapy?

Both ion channels have shown to regulate recycling of membranous proteins (E-cadherin and β1-integrin) in cancer cells favoring tumor progression (33, 57, 74). TPC2 is known to regulate recycling processes of β1-integrin in cancer cells, as ablation leads to attenuation in early endosomes (57). Very recent research has shown that TRPML1 mediates trafficking of E-cadherin to the plasma membrane by altering the expression of trafficking markers Rab11 or Rab5 in cancer (74). In addition, in macrophages TRPML1 has been shown to be required for the efficient transport of MHC-II complex molecules to the plasma membrane (97). Despite their undeniable influence on cellular trafficking and the expression of Rab proteins, the precise mechanisms through which TRPMLs and TPCs regulate the trafficking of receptors and membrane proteins have remained elusive so far. To shed light on these mechanisms, we propose investigating direct endolysosomal patch clamps of GTPases such as Rab5, Rab11, and Rab7, with a focus on protein-protein interactions.

In 2023, Davis et al. introduced a novel hypothesis suggesting that calcium nanodomains may be instrumental in ion channel-specific trafficking (188). They proposed a potential explanation for why TPC2 fails to induce lysosomal exocytosis during phagocytosis: the calcium it releases may not effectively reach the exocytotic machinery, which is presumed to be adjacent to TRPML1. They put forward a model wherein calcium released from lysosomal calcium channels does not create a continuous ‘halo’ of high calcium concentration around the lysosome, but rather forms a discontinuous ‘mosaic’ of calcium nanodomains associated with each channel point-source (188). While intriguing, their hypothesis requires validation in diverse cell lines and models. Consequently, we advocate for future research focusing on calcium nanodomains to elucidate precise molecular mechanisms for TRPMLs and TPCs.

In summary, this review highlights that despite significant progress in understanding the involvement of TRPMLs and TPCs in immunity, many unanswered questions persist regarding their role in cancer immunity. Specifically, TRPML1 exhibits numerous connections in maintaining the TME or aiding in immune evasion. However, a far-reaching study on its effect in cancer immunity is missing. Also, the precise role of TPC2 in this context remains an area that requires elucidation in future research. So far, there is a lack of knowledge on the role of both families of ion channels on the development of a pro-tumorigenic and pro-inflammatory TME and immune response, including the development of TAMs. What is the role of TPCs and TRPMLs in the regulation of cytokine secretion in macrophages and/or cancer cells? What are their roles in modulating MHC molecule and antigen presentation for proper T cell activation? Would the targeting of these ion channels present a dual role in treating cancer? Consequently, these channels may hold potential explanations or key contributions to the described mechanisms enabling cancer cells to evade immune destruction, uphold a resilient TME and reduce activation of T cells.

## 5 Conclusion

Here we summarize the current knowledge on the role of TRPMLs and TPCs in cancer development and in regulatory mechanisms in innate and adaptive immune response (**Table 1**). Both families of ion channels have shown to play key roles in the process of phagocytosis and immune cell signaling. Additionally, TRPMLs act as regulators between innate and adaptive immune system by secreting chemokines and regulating MHC-I expression. Due to rising resistance mechanisms in cancer immunotherapy, we emphasize the great potential that lays in elucidating research gaps in cancer immunity, especially mechanisms that aid evading immune destruction and surveillance.

**Table 1:** TPCs and TRPMLs in immunity and their potential effect on cancer immunity.

Ion channel	Finding	Hypothesis – research gap	Reference
**TRPML1**	Required for the efficient exit of MHC-II and MHC-I molecules destined for the plasma membrane in macrophages. Reduces MHC-I expression on pancreatic cancer.	Lysosomal degradation of MHC-I observed in pancreatic cancer is regulated by TRPML1-mediated autophagy, subsequently leading to reduced anti-tumor CD8+ T cell reaction.	(33, 97)
**TRPML2**	Promotes the release of major cytokine CCL2 from macrophages	Recruitment and polarization of macrophages to M2 macrophages in the TME What other cytokine releases are mediated through TRPMLs that contribute to an inflammatory TME?	(95)
**TPC1**	Regulation of exocytosis event of histamine secretion in mast cells	TPCs may play a role in lysosomal exocytosis in immune cells. Do they also affect cytokine release in the TME?	(121, 189)
**TPC2**	Key element in controlling inflammatory leukocyte recruitment and adhesion via P-Selectin Blockage of TPC2 in BMDMs results in reduced activation of the immune system, due to endolysosomal trapping.	Evading immune destruction: P-Selectin complex forming and thereby masking tumor cells from recognition by macrophages What other cytokine releases are mediated through TRPMLs and TPCs that contribute to an inflammatory TME?	(116, 117) (115)
**General**	CTLA-4 and PD-L1 expressed on cancer cells, contribute to immune evasion. Their expression and their recycling, has been implicated in being regulated through the ES	TPCs and TRPMLs regulate expression of membrane proteins. Do TPCs and TRPMLs regulate expression of immune checkpoints on cancer cells?	(33, 57, 74)

All the discussed findings of TPCs and TRPMLs and their potential effect on cancer immunity.

## Author contributions

LO: Writing – review & editing, Writing – original draft. KB: Conceptualization, Funding acquisition, Writing – review & editing.
